# Cough in the Elderly Population: Relationships with Multiple Comorbidity

**DOI:** 10.1371/journal.pone.0078081

**Published:** 2013-10-21

**Authors:** Woo-Jung Song, Alyn H. Morice, Min-Hye Kim, Seung-Eun Lee, Eun-Jung Jo, Sang-Min Lee, Ji-Won Han, Tae Hui Kim, Sae-Hoon Kim, Hak-Chul Jang, Ki Woong Kim, Sang-Heon Cho, Kyung-Up Min, Yoon-Seok Chang

**Affiliations:** 1 Department of Internal Medicine, Seoul National University College of Medicine, Seoul, Korea; 2 Institute of Allergy and Clinical Immunology, Seoul National University Medical Research Center, Seoul, Korea; 3 Cardiovascular and Respiratory Studies, Hull York Medical School, Castle Hill Hospital, University of Hull, Cottingham, East Yorkshire, United Kingdom; 4 Department of Internal Medicine, Seoul National University Bundang Hospital, Seongnam, Gyeonggi-do, Korea; 5 Department of Internal Medicine, Gachon University Gil Medical Center, Incheon, Korea; 6 Department of Neuropsychiatry, Seoul National University Bundang Hospital, Seongnam, Gyeonggi-do, Korea; 7 Department of Psychiatry, Seoul National University College of Medicine, Seoul, Korea; Kliniken der Stadt Köln gGmbH, Germany

## Abstract

**Background:**

The epidemiology of cough in the elderly population has not been studied comprehensively. The present study aimed to investigate the epidemiology of cough in a community elderly population, particularly in relation with their comorbidity.

**Methods:**

A cross-sectional analysis was performed using a baseline dataset from the Korean Longitudinal Study on Health and Aging, a community-based elderly population cohort study. Three types of cough (frequent cough, chronic persistent cough, and nocturnal cough) were defined using questionnaires. Comorbidity was examined using a structured questionnaire. Health-related quality of life was assessed using the Short Form 36 questionnaire.

**Results:**

The prevalence was 9.3% for frequent cough, 4.6% for chronic persistent cough, and 7.3% for nocturnal cough. In multivariate logistic regression analyses, smoking, asthma and allergic rhinitis were found to be risk factors for cough in the elderly. Interestingly, among comorbidities, constipation and uncontrolled diabetes mellitus (HbA1c ≥ 8%) were also found to have positive associations with elderly cough. In the Short Form 36 scores, chronic persistent cough was independently related to impairment of quality of life, predominantly in the mental component.

**Conclusions:**

Cough has a high prevalence and is detrimental to quality of life in the elderly. Associations with smoking, asthma and rhinitis confirmed previous findings in younger populations. Previously unrecognised relationships with constipation and uncontrolled diabetes mellitus suggested the multi-faceted nature of cough in the elderly.

## Introduction

Cough is a fundamental defensive reflex protecting against aspiration into the airways. The reflex can be up-regulated by a number of irritants such as acute respiratory viral infections, which are mostly self-limited. However, some individuals experience a prolonged cough, lasting for several weeks or longer. 

It has been suggested that chronic cough results mostly from the disease triad of asthma, rhinosinusitis, and gastroesophageal reflux disease (GERD) [1]. For decades, this paradigm has been used in the management of chronic cough [2]. However, recently doubt regarding its generality has been expressed [3-5]. Clinicians are confronted with many patients with chronic cough who do not easily fit into any diagnostic category within the classical paradigm. In this context, it was proposed that chronic cough should be re-appraised as an independent single disease entity with intrinsic pathophysiology of cough reflex hypersensitivity [5,6].

These new concepts into the pathogenesis of cough require a re-evaluation of the epidemiological features and disease associations seen in subjects with cough. Various conditions may directly or indirectly affect physiological function leading to regulation of the cough reflex. This is particularly important in the elderly population, as they often have multiple comorbidities. To date, no epidemiologic survey has comprehensively examined the potential associations between comorbid illnesses and cough in the elderly. This has important clinical consequences, as the majority of cough patients are middle-aged or elderly persons [5,7].

The Korean Longitudinal Study on Health and Aging (KLoSHA) is a community population-based study of persons aged ≥65 years old. It was designed as a prospective cohort study to provide comprehensive data on common disorders in the elderly population assessed by multi- and inter-disciplinary approaches. Using the baseline cross-sectional KLoSHA dataset, we have examined the epidemiology of cough and explored the importance of associated comorbidities in this well-defined elderly population. 

## Materials and Methods

### Study population

This analysis was a part of the baseline KLoSHA survey, which has been previously described in detail [8]. Briefly, the study was conducted from September 2005 to August 2006. A total of 1,000 elderly participants (aged 65+ years) were recruited from among the residents of Seongnam, a major satellite city of Seoul, Republic of Korea. The total population of Seongnam was 977,166 in 2004, and the proportion of elderly persons was 6.2%. They were selected using a stratified random sampling method and were invited by post and then by telephone to participate. Of the 1,118 individuals who were randomly selected, 698 agreed to participate. To examine the health status of the oldest persons (those aged 85+ years), all of the oldest residents were additionally contacted by letter and telephone, and 302 agreed to participate. The present analysis was undertaken on the 796 subjects who completed all the cough-related questionnaires and assessments. All the interviews were performed by trained nurses with certifications in epidemiologic studies and geriatric assessments. All assessments were performed at Seoul National University Bundang Hospital. All participants were fully informed of the protocol and provided written statements of informed consent. The protocol was approved by the institutional review board of Seoul National University Bundang Hospital.

### Assessments

The current medical history was checked by trained interviewers using a structured questionnaire containing questions of the following type: “Have you been diagnosed with or treated for (a specific disease) by a physician in the last 12 months?” The questionnaire items included disease categories by organ and detailed questions about the presence of more than 70 specific diseases that are common in the Korean elderly population (Table S1). Participants were also asked to bring current medications or prescriptions. The total burden of chronic illness was rated using the modified Cumulative Illness Rating Scale (CIRS) [9]. Health-related quality of life (HRQoL) was measured using the Korean version of the Short Form 36 (SF-36) Health Survey [10].

The prevalence of cough was defined by the use of a modified Korean language version of the International Union Against Tuberculosis and Lung Disease (IUATLD) and the American Thoracic Society and Division of Lung Diseases of the National Heart and Lung Institute (ATS-DLD-78-adult) questionnaires [11]. *Frequent cough* was defined by the question “Do you usually cough as much as four to six times a day, four or more days a week?”, and *chronic persistent cough* was defined by positive answer to the subsequent question “Do you usually cough like this on most days for three consecutive months or more during the year?” *Nocturnal cough* was assessed by the question “Have you been wakened by an attack of coughing at any time in the last 12 months?”

Height and weight were measured to the nearest 0.1 cm and 0.1 kg, respectively. Body mass index (BMI) was defined as weight divided by height squared. Spirometry was performed by an experienced technician using a portable spirometer (Vmax-2130; SensorMedics, Yorba Linda, CA) according to the criteria of the ATS[12]. Predicted values of forced expiratory volume in 1 second (FEV1) and forced vital capacity (FVC) were obtained using the methods described by Morris [13]. Serum hemoglobin A1C (HbA1c) levels were measured by high-performance liquid chromatography (HPLC) using a Variant II HPLC system (Bio-Rad Laboratories, Hercules, CA). Chest radiographs were obtained, and the chest radiograph abnormality was defined as positive if the subject had bronchiectasis, emphysema, tuberculosis, malignancy, or any other grossly abnormal parenchymal lesions in the interpretations by two independent radiologists.

### Statistical analysis

For descriptive statistics, continuous variables are presented as the mean values and standard deviation, and categorical variables as frequencies. Differences between groups were evaluated by Student’s *t*-test or Mann–Whitney *U*-test for continuous variables and by the chi-square test for categorical variables. Logistic regression was performed to explore factors associated with cough. Controls were defined as subjects without any of the three types of cough. To determine the independent impact of cough on HRQoL, a general linear model with adjustment for confounders with *p* values <0.1 in univariate tests was used. All statistical tests were performed using Stata software package (release 12.0; Stata Corp., Texas, USA). All statistical tests were two-sided, and *p* values <0.05 were considered statistically significant. 

## Results

### Baseline characteristics

**Figure 1:**
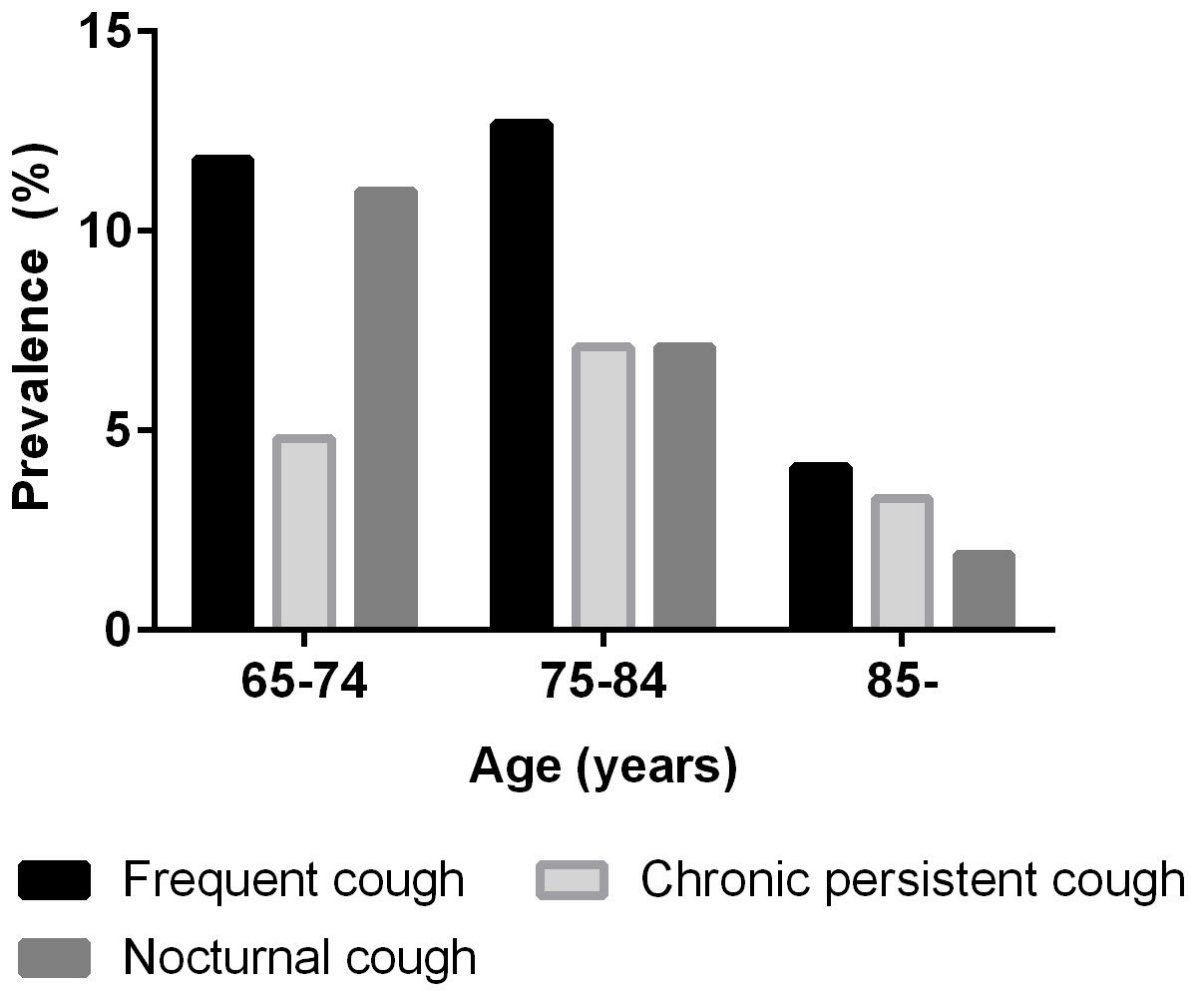
Prevalence of cough according to age groups.

The baseline characteristics of the study population are shown in Table 1. The prevalence of cough was 9.3% for frequent cough, 4.6% for chronic persistent cough, and 7.3% for nocturnal cough. Female subjects had more frequent nocturnal cough than males (9.3% vs. 4.9%; *p*= 0.018); however, no significant gender difference was found for frequent cough (10.4% vs. 8.0%; *p*= 0.246) or chronic persistent cough (5.2% vs. 4.0%; *p*= 0.423). When classified by age groups, the prevalence of cough significantly decreased with aging (for nocturnal cough, *p* for linear-by-linear associations< 0.001; and for frequent cough, *p*= 0.001), except for chronic persistent cough (*p*= 0.451; Figure 1). 

**Table 1:** Baseline characteristics of the study population.

Variable	Frequent cough (n= 74)^*†^	Chronic persistent cough (n= 37)^*‡^	Nocturnal cough (n= 58)^*§^	Control (n=688) ^||^
Age (years)	74.2 ± 8.0^††^	75.9 ± 8.2	72.0 ± 6.8^‡‡^	77.5 ± 9.2
Male gender (%)	37.8	37.8	29.3^**^	45.0
BMI (kg/m^2^)	23.6 ± 3.3	22.7 ± 2.9^**^	24.9 ± 3.3^**^	23.9 ± 3.3
Smoking (%)				
Never smoker	59.5^**^	56.8^**^	68.4	62.3
Ex-smoker	18.9	18.9	22.8	26.8
Current smoker	21.6	24.3	8.8	10.8
Alcohol (%)				
Never drinker	64.4	61.1	65.5	60.8
Ex-drinker	16.4	19.4	22.4	15.5
Current drinker	19.2	19.4	12.1	23.7
Education (years)	7.2 ± 5.0	5.5 ± 5.3	6.3 ± 5.8	7.4 ± 5.6
Marriage				
Married	62.2	64.9	62.1	69.3
Never married/divorced/bereaved	37.8	35.1	37.9	30.7
Income				
>12,000 US dollars/year	46.3	45.9	46.6	45.9
≤12,000 US dollars/year	53.7	54.1	53.4	54.1
Regular exercise (%)	50.0	45.9	50.0	50.0
ACE inhibitor use (%)	2.7	5.4	0.0	1.9
CIRS-T	3.7 ± 2.0	3.9 ± 1.9	4.1 ± 2.5	3.8 ± 2.5
Spirometry				
FEV1, % predicted	103.2 ± 22.3	102.1 ± 25.9	108.0 ± 19.2	106.5 ± 24.9
FVC, % predicted	94.0 ± 15.8	91.7 ± 17.2	96.3 ± 13.9	94.3 ± 18.6
FEV1/FVC, %	74.0 ± 10.8	74.9 ± 11.0	75.1 ± 7.1	76.1 ± 9.2
FEV1/FVC <70% (%)	14.9	13.5	15.5	11.8
Chest radiograph abnormality^¶^ (%)	9.5^**^	13.5	10.3	19.3

Abbreviations: BMI, body mass index; GERD, gastroesophageal reflux disease; ACE, angiotensin-converting enzyme; CIRS-T, total score for cumulative illness rating scale; FEV1, forced expiratory volume in 1 second; FVC, forced vital capacity

* Different types of cough are not mutually exclusive.

† Frequent cough was defined by the response to the following question: “Do you usually cough as much as four to six times a day, 4 or more days a week?”

‡ Chronic persistent cough was defined by the response to the following question: “Do you usually cough like this on most days for 3 consecutive months or more during the year?”

§ Nocturnal cough was defined by the response to the following question: “Have you been woken by an attack of coughing at any time in the last 12 months?”

|| Control group was defined by negative responses to all of the three types of cough.

¶ Chest radiograph abnormality was defined as positive if the subject had bronchiectasis, emphysema, tuberculosis, malignancy, or any other grossly abnormal parenchymal lesions.

^**^ *p*< 0.05, ^††^ *p*< 0.01, and ^‡‡^ *p*< 0.001; Differences between coughers and controls were assessed by Student’s *t*-test or Mann–Whitney *U*-test for continuous variables and by the chi-square test for categorical variables.

Various clinical factors were explored for their relationships with cough. Among comorbidity conditions, asthma, allergic rhinitis, diabetes mellitus, constipation, and gastritis showed potential associations (*p*<0.1; Table 2); thus they were assessed further for cough relationships in multivariate logistic regression analyses. However, airway obstruction (defined as an FEV1/FVC of <70%), or chest radiograph abnormality did not have positive associations with any type of cough.

**Table 2:** Frequency of comorbid conditions in each type of cough.

	Frequent cough, n (%)	Chronic persistent cough, n (%)	Nocturnal cough, n (%)	Controls^§^, n (%)
Asthma	6/74 (8.1%)^*^	6/37 (16.2%)^†^	9/57 (15.8%)^‡^	21/686 (3.1%)
Allergic rhinitis	6/74 (8.1%)	5/37 (13.5%)^*^	6/58 (10.3%)^*^	30/687 (4.4%)
Stroke	4/73 (5.5%)	2/37 (5.4%)	3/56 (5.4%)	21/673 (3.1%)
Parkinson’s disease	1/70 (1.4%)	1/35 (2.9%)	0/57 (0%)	9/641 (1.4%)
Hypertension	36/70 (51.4%)	18/36 (50.0%)	31/53 (58.5%)	300/665 (45.1%)
Diabetes mellitus				
Diabetes mellitus	17/74 (23.0%)^*^	7/37 (18.9%)	13/58 (22.4%)^*^	90/688 (13.1%)
HbA1C ≥ 8%	8/74 (10.8%)^†^	3/37 (8.1%)	5/58 (8.6%)^*^	19/684 (2.8%)
Constipation	19/74 (25.7%)^*^	17/37 (45.9%)^‡^	11/58 (19.0%)	107/688 (15.6%)
IBS	4/74 (5.4%)	3/37 (8.1%)	4/58 (6.9%)	31/688 (4.5%)
Gastritis	13/73 (19.2%)	8/37 (21.6%)^*^	10/58 (17.2%)	69/666 (10.4%)
GERD	2/70 (2.9%)	1/36 (2.8%)	0/54 (0%)	7/684 (1.0%)

Abbreviations: IBS, irritable bowel syndrome; GERD, gastroesophageal reflux disease

* *p*< 0.05; ^†^ *p*< 0·01; and ^‡^ *p*< 0.001: Differences between coughers and controls were determined by chi-squared tests.

For definitions, see Table 1.

§ Control group was defined by negative responses to all of the three types of cough.

### Conditions associated with cough

Multivariate logistic regression was used to more closely identify the conditions associated with cough (Tables 3 and 4). In Table 3, age, gender, BMI and smoking were selected as independent variables; and in Table 4, any positively related comorbid conditions (*p*< 0.1) in univariate tests were further included for adjustment. Considering the possibility of multiple testing errors, the corrected significance level was determined as *p*< 0.007 (=0.05/7; seven variables from six comorbid conditions; asthma, allergic rhinitis, smoking status, diabetes mellitus/’HbA1c ≥8%’, constipation and gastritis).

**Table 3:** Adjusted logistic regression models for cough and comorbidity relationships.

	Frequent cough		Chronic persistent cough		Nocturnal cough	
	Adjusted OR (95% CI)^*^	*p* value	Adjusted OR (95% CI)^*^	*p* value	Adjusted OR (95% CI)^*^	*p* value
Age (years)	0.95 (0.92–0.99)	0.009	0.97 (0.92–1.01)	0.117	0.91 (0.87–0.96)	<0.001
BMI (kg/m^2^)	0.95 (0.88–1.04)	0.268	0.90 (0.80–1.01)	0.085	1.06 (0.96–1.16)	0.258
Male gender	0.43 (0.21–0.87)	0.019	0.41 (0.16–1.04)	0.060	0.44 (0.19–1.05)	0.065
Smoking status						
Ex-smoker	1.29 (0.55–3.02)	0.565	1.47 (0.48–4.49)	0.495	1.32 (0.49–3.56)	0.590
Current smoker	3.79 (1.76–8.18)	0.001	3.83 (1.42–10.3)	0.008	1.30 (0.42–3.99)	0.647
Asthma	2.99 (1.09–8.22)	0.034	6.16 (2.16–17.6)	0.001	5.33 (1.86–15.3)	0.002
Allergic rhinitis	2.19 (0.84–5.73)	0.108	3.85 (1.31–11.3)	0.014	3.04 (1.14–8.08)	0.026
Diabetes mellitus	2.50 (1.33–4.72)	0.005	2.01 (0.82–4.97)	0.130	1.73 (0.84–3.56)	0.139
HbA1C ≥ 8%	4.15 (1.90–9.07)	<0.001	6.55 (1.64–26.2)	0.008	3.95 (1.24–12.6)	0.020
Constipation	2.48 (1.29–4.77)	0.006	5.97 (2.76–12.9)	<0.001	2.28 (1.00–5.16)	0.049
Gastritis	1.72 (0.86–3.44)	0.123	2.21 (0.93–5.25)	0.074	1.69 (0.78–3.66)	0.183

Abbreviations: BMI, body mass index; OR, odds ratio; CI, confidence interval

For definitions, see Table 1.

* *P* values were determined by multivariate logistic regression tests with adjustment for age, gender, BMI and smoking status.

**Table 4:** Further adjusted logistic regression models for cough and comorbidity relationships.

	Frequent cough		Chronic persistent cough		Nocturnal cough	
	Adjusted OR (95% CI)^*^	*p* value	Adjusted OR (95% CI)^†^	*p* value	Adjusted OR (95% CI)^*^	*p* value
Age (years)	0.95 (0.91–0.98)	0.003	0.94 (0.90–0.99)	0.026	0.90 (0.85–0.94)	<0.001
BMI (kg/m^2^)	0.94 (0.86–1.03)	0.171	0.91 (0.80–1.03)	0.144	1.06 (0.96–1.17)	0.288
Male gender	0.39 (0.19–0.81)	0.011	0.44 (0.16–1.22)	0.113	0.39 (0.16–0.95)	0.037
Smoking status						
Ex-smoker	1.32 (0.55–3.15)	0.537	1.42 (0.44–4.56)	0.557	1.45 (0.53–3.98)	0.469
Current smoker	4.22 (1.91–9.39)	<0.001	4.10 (1.37–12.3)	0.012	1.37 (0.43–4.35)	0.589
Asthma	2.89 (0.99–8.42)	0.052	5.94 (1.80–19.6)	0.003	5.70 (1.95–16.7)	0.001
Allergic rhinitis	2.51 (0.93–6.77)	0.068	5.29 (1.60–17.5)	0.006	2.71 (0.91–8.07)	0.074
HbA1C ≥ 8%	10.0 (3.68–27.2)	<0.001	11.0 (2.56–47.6)	0.001	5.10 (1.58–16.5)	0.006
Constipation	2.64 (1.33–5.23)	0.005	6.84 (2.87–16.3)	<0.001	2.74 (1.18–6.37)	0.020
Gastritis			1.69 (0.65–4.42)	0.284		

Abbreviations: BMI, body mass index; OR, odds ratio; CI, confidence interval

For definitions, see Table 1.

*P* values were determined by multivariate logistic regression tests.

* Adjusted for age, gender, BMI, smoking status, asthma, allergic rhinitis, HbA1C ≥ 8%, and constipation

† Adjusted for age, gender, BMI, smoking status, asthma, allergic rhinitis, HbA1C ≥ 8%, constipation, and gastritis

First, frequent cough was positively related with current smoking, diabetes mellitus (or ‘HbA1c ≥8%’) and constipation. ‘HbA1c ≥8%’ was more strongly related (odds ratio [OR] 4.15, 95% confidence interval [CI] 1.90–9.07, *p*< 0.001), compared to diabetes history (OR 2.50, 95% CI 1.33–4.72, *p*= 0.005). Therefore, ‘HbA1c ≥8%’was selected as the independent variable for further adjusted models (Table 4), instead of diabetes history. As results, the positive relationships of following conditions were identified: current smoking, ‘HbA1c ≥8% and constipation. 

Chronic persistent cough had positive associations with current smoking, asthma, allergic rhinitis, ‘HbA1c ≥8%’, and constipation. Their positive associations (asthma, allergic rhinitis, ‘HbA1c ≥8%’, and constipation) remained significant in the further adjusted models. Meanwhile, nocturnal cough showed significant association only with asthma, and marginally with ‘HbA1c ≥8%’. 

In order to confirm the lack of influence of chest radiograph abnormality and current medications, we repeated the multivariate logistic regression tests after excluding the subjects with abnormal radiograph findings (n=143) and those taking ACE inhibitors (n=16) or codeine (n=3), and found that the overall conclusions remained unchanged (Table S2). 

### Cough-related impairment of quality of life

In univariate analyses, cough showed significant reductions in the scores of SF-36 scales (Table 5); and particularly, chronic persistent cough had more impairments than other forms of cough. To test independent relationships between chronic persistent cough and quality of life, general linear models were used with adjustment for comorbidity burden (CIRS) and demographic variables with *p*-values <0.1 (gender, marriage status, and income). As results, chronic persistent cough was independently associated with reduction in the scales including ‘bodily pain’, ‘mental health’, and ‘mental component summary’ score. Chronic persistent cough was found to have reduced ‘mental component summary’ score (46.8±9.5) lower than that of stroke (48.5±10.6) or Parkinson’s disease (49.1±8.5). 

**Table 5:** SF-36 HRQoL questionnaire scores according to the presence of cough.

	Frequent cough	Chronic persistent cough	Nocturnal cough	Controls
Each scale				
General health	39.0 ± 22.9	36.8 ± 22.2^†^	36.5 ± 22.4^‡^	44.2 ± 21.5
Physical functioning	48.4 ± 25.5	47.6 ± 23.8	48.3 ± 27.0	54.5 ± 29.2
Role limitation due to physical problem	60.0 ± 31.9^‡^	59.6 ± 29.0^†^	60.0 ± 33.6^†^	68.9 ± 30.8
Role limitation due to emotional problem	75.8 ± 27.0^†^	71.1 ± 29.9^†^	77.6 ± 28.0	79.8 ± 28.4
Social functioning	73.7 ± 27.0	72.4 ± 24.4	74.6 ± 24.1	78.8 ± 24.6
Bodily pain	52.3 ± 28.3^‡^	46.4 ± 26.6^‡^	53.7 ± 29.0^†^	62.9 ± 29.6
Vitality	48.9 ± 20.2	45.6 ± 19.2^†^	46.7 ± 21.3	52.4 ± 20.7
Mental health	62.2 ± 21.7^†^	57.5 ± 20.6^‡^	65.4 ± 17.5	68.8 ± 19.9
Summary score				
PCS	35.6 ± 10.6^†^	35.0 ± 9.1^†^	35.0 ± 11.6^†^	38.7 ± 10.8
MCS	48.9 ± 10.3	46.8 ± 9.5^†^	49.8 ± 7.1	50.8 ± 9.2

Abbreviations: HRQoL, health-related quality of life; PCS, physical component summary; MCS, mental component summary

For definitions, see Table 1 and 2. P values were determined by Mann–Whitney U-tests; ^†^ *p*< 0.05 (versus controls); ^‡^ *p*< 0.01 (versus controls)

## Discussion

In the present study, we have investigated the prevalence of cough and associated conditions in a well-defined elderly community population. We have shown the expected associations with current smoking, asthma, and allergic rhinitis with cough. Interestingly, we also found previously unrecognised associations with constipation and uncontrolled diabetes mellitus (HbA1c ≥8.0%). We have additionally demonstrated that chronic persistent cough was detrimental to the quality of life of the elderly persons. 

Cough is one of the most common respiratory conditions seeking medical attention [14]. Importantly, a majority of chronic cough patients seeking a specialized cough clinic is middle-aged or elderly persons [15]. However, in the literature so far, the cough in older adults has not been characterized in clinical or epidemiological studies. Considering the rapid aging of global population [16], cough in the elderly should be investigated for its unique characteristics such as prevalent comorbid conditions. As expected, we found that comorbid conditions were frequent in the elderly, showing an average score of 3.8 in CIRS. This issue of high comorbidity may have been a reason for the lack of previous studies for elderly population. In this regard, the present study has unique strength that it identified the epidemiology of elderly cough, using a well-defined elderly population cohort with structured questionnaire items for comorbidity. 

To our knowledge, it is the first report that constipation is an independently associated condition in elderly subjects with cough. Chronic idiopathic constipation or irritable bowel syndrome with constipation (IBS-C) has been examined for various comorbid conditions, but has rarely been investigated for respiratory comorbidities, and moreover not for cough [17]. In contrast to the well-described association of upper gastrointestinal symptoms with cough, the relationship of lower bowel symptoms has not been elucidated. With regard to the mechanism of association, firstly, we presume that it could be a direct effect of constipation itself. For instance, elderly persons with constipation may have increased abdominal pressure resulting in an increased incidence of gastroesophageal reflux, combined with a decrease in oesophageal peristalsis or sphincter functions [18]. Cough and constipation have characteristics in common, with a high prevalence in aged female subjects [17,19,20] and being regulated by the vagus nerve [21,22]. Since different aetiologies of constipation exist [19], further analysis may identify the mechanisms of association with chronic cough. 

The relationship of gastrointestinal symptoms with cough has been previously addressed in a community population survey in the UK adult population (aged 50–59 years) [23], that identified a significant relationship between chronic cough and irritable bowel syndrome (IBS) using the Manning criteria [24]. Due to the difference in methodology and demographics, two epidemiologic findings are not directly comparable. However, considering that constipation is a frequent gastrointestinal condition becoming increasingly prevalent with aging [19], the relationships between chronic cough and gastrointestinal disorders warrant further characterization. 

GERD is known as a risk factor for chronic cough. However, we failed to find an association between cough and GERD. This potential underestimation could be related to our eliciting the history of GERD by ‘current diagnosis or treatment by a physician’ rather than specific symptoms. We found low rates of current GERD diagnosis in our population (1·1% vs. 14·6–22·9% in the UK survey [23]). In the UK survey, the association with cough of peptic reflux symptoms such as indigestion and heartburn was much less than the other classic, but non-acid related symptom, of regurgitation. This observation, coupled with the failure of antacid therapy to inhibit cough [25], has led to the suggestion that the component of reflux responsible for cough is non-acid in nature and therefore unlikely to have been observed in our study which relied on physician diagnosed GERD. When specific questionnaires, designed to detect cough-related reflux, have been administered, the prevalence of reflux is higher and peptic symptoms are poorly predictive [5].

The association between poorly controlled diabetes mellitus and elderly cough is another novel finding. Patients with diabetes mellitus have a higher prevalence of GER [26] or laryngopharyngeal reflux [27] than their non-diabetic counterparts. A possible mechanism is diabetic neuropathy, as the presence of neuropathy has been associated with abnormal oesophageal [28,29] or laryngopharyngeal reflux [27], leading to a reflux cough [30]. It may appear confusing, as the diabetic neuropathy was also associated with decreased cough reflex sensitivity to peripheral capsaicin or citric acid challenges [31,32]. Therefore, the mechanisms of association warrant further studies. We speculate that the mechanisms could include the pathophysiological changes increasing reflux risks, or possibly also be related to central sensitization. The symptoms suggestive of laryngeal paresthesia were particularly more frequent in diabetic patients [27]. In cases for pain, which are recently suggested to have similar pathophysiological aspects with cough hypersensitivity, poor diabetes control has been associated with decreased pain threshold [33]. Our relationships with high HbA1C levels further suggest diabetes mellitus, especially uncontrolled, needs to be considered as a potential risk factor for cough, particularly in the elderly population. There is still a paucity of studies investigating the associations between HbA1c and non-diabetic, or non-cardiovascular conditions; however, high HbA1c was positively related to reflux symptom episodes in adolescents [34], and also was independently associated with the increased prevalence of pain [35,36].

It may be argued that diabetic subjects are more likely to receive angiotensin converting enzyme inhibitors (ACE-i) and thus to be at greater risk of cough. However, in our analyses, current medication history was also obtained, and the possible confounding effects were minimised. The prescription rate of ACE-i of 4.5% in the present population was lower than 11.7% in the Korean National Health Insurance claims database analyses during the same period [37]; however, the prescription may be influenced by primary indications or socioeconomic status. Calcium channel blockers (64.4%), diuretics (44.6%), and angiotensin receptor blockers (33.3%) had higher prescription rates in Korean populations [37]. In addition, we would like to mention the associations between diabetes mellitus and chronic idiopathic constipation, or IBS with constipation [17]. Collectively, despite several limitations in outcome measurements in the present study, we suggest that potential roles of diabetes mellitus on cough should be further studied. 

Our findings on allergic rhinitis are consistent with previous surveys in general population [20,38], and are in line with the perspective of the American College of Chest Physicians, which considers upper airway cough syndrome to be one of the most common causes of chronic cough [39]. Asthma is also a well-known association of cough in the general population [20,38], which was also confirmed for elders in the present study. The positive association with current smoking has been also reported previously [40]. 

Age and gender are important demographic factors related to cough. Our results showed an inverse relationship with ageing, particularly for nocturnal or frequent cough. There is some evidence that the cough reflex decreases in elderly populations, particularly in those with cognitive impairment [41]. Female predominance is well-recognised in adult cough patients [5]. Our finding that the gender difference was less prominent in older persons could be aging effects, as similar findings were observed in a Swedish population study where female preponderance was observed in subjects who were 40–70 years old but not in those aged over 70 years [20].

As the present study was conducted in the elderly population, it could not compare with younger adults. The prevalence of cough appears to vary considerably between populations [40], possibly due to environmental or methodological differences. The European Community Respiratory Health Survey found the prevalence of nocturnal cough as 30.7% in European adults aged 20–48 years old [38], which was much higher than 7.3% in the present survey using the same questionnaire item. However, another recent general population survey, the Atherosclerosis Risk in Communities study, found the prevalence of frequent cough (originally defined as chronic cough in their study) as 9.7% in American adults aged 45–64 years old [42], which was quite similar to our findings of 9.3% in the Korean elders. The prevalence of chronic persistent cough was also similarly reported as 4.1% in Italian adults aged 18–60 years old who participated in the SAPALDIA cohort [43]. Unlike nocturnal or frequent cough, the present study found that chronic persistent cough did not significantly decrease with aging, which could suggest significant clinical burdens from chronic persistent cough across various ages.

The impact of chronic cough on health-related quality of life is a well-known, but poorly appreciated finding [44,45]. In the present study, using the SF-36 questionnaire, we demonstrated that chronic persistent cough independently impairs quality of life in the elderly, predominantly in the mental aspects. The degree of mental health impairments in chronic persistent cough was more severe than in stroke or Parkinson’s disease. These findings suggest that chronic cough in the elderly is a major hidden health problem that requires further investigation. 

The current study has several limitations. It has a cross-sectional design and thus could not address causal relationships with comorbidity. Lack of statistical power and precision from a small sample size is a major limitation, which derives from the original cohort design. The KLoSHA study was designed for various common geriatric disorders by multidisciplinary approaches [8], enabling the association studies. In addition, selection bias needs to be taken into consideration, as a sizeable group (420 subjects from 1,118 randomly selected persons) refused to participate during the initial recruitment step. A possibility of recall bias also needs to be considered, as the outcome measurement was based on questionnaires for symptoms and medical history. Our lack of objective or detailed measurements for cough reflex and comorbidities (i.e., reflux, types of diabetes mellitus, or diabetic complications) was also a limitation, necessitating further validation studies.

Definitions of cough may also be a limitation, as currently available questionnaires on cough were originated from the general respiratory questionnaires for asthma or chronic bronchitis (cough and/or phlegm) [11]. However, in the literature so far, no standard questionnaires have been designed or validated for the cough epidemiology itself. Moreover, considerable heterogeneity can be found for the cough definitions or questions utilized in previous epidemiologic studies [40]. For example, chronic cough was sometimes defined by the question ‘Do you usually cough as much as four to six times a day, four or more days a week?’ [42], but also by ‘Do you usually cough like this on most days for three consecutive months or more during the year?’ in other studies [46,47]. Similarly, the related question items have been also utilized for assessing ‘habitual cough’[48], ‘persistent cough’ [49], or ‘usual cough’ [46]. The problem is also related to the discrepancy in defining the duration for chronic cough (8 weeks in the clinical guidelines [2,50] vs. 3 months in the epidemiological studies). Thus, we determined to assess them by calling ‘frequent cough’ and ‘chronic persistent cough’. Along with recent advances in understanding cough hypersensitivity [5,6], chronic cough has just begun to be considered as a disease entity with intrinsic pathophysiology. Collectively, we feel that it would be an essential step to develop the standard questionnaires for cough epidemiology surveys.

The present study has strength: it is a comprehensive analysis of a well-defined elderly community population, using structured questionnaire items on various comorbid conditions and medication. Another strength is that more than 25% of participants were ‘oldest’ persons (those aged 85+ years), who have rarely been included in previous studies. Our findings of previously unrecognised associations could have been attributed to the inclusion of the older subjects. Furthermore, our utilization of objective measures such as spirometry and chest radiograph is additional strength. Finally, despite several limitations discussed above, we expect that our explorative analyses could provide clues for further studies. 

In conclusion, cough was prevalent, and significantly associated with quality of life impairments in the elderly population. We found previously unrecognised positive associations with constipation and uncontrolled diabetes mellitus. These findings may indicate the multi-faceted nature of elderly cough. Further studies will be required to confirm these associations and explore their causal relationships.

## Supporting Information

Table S1 **List of comorbidity.**
(DOC)

Table S2 **Logistic regression models for cough and comorbidity relationships among the subjects without abnormal chest radiographs and ACE inhibitor/codeine medication.**
(DOC)
