# Supplementary material for: Cough in the Elderly Population: Relationships with Multiple Comorbidity
Source: PLoS One. 2013 Oct 21;8(10):e78081. doi: 10.1371/journal.pone.0078081 (PMC3804463; doi:10.1371/journal.pone.0078081)
Supplement: Table S1 — List of comorbidity. (DOC) [file pone.0078081.s001.doc]

**Supporting information**

**Appendix Table**

Table S1. List of comorbidity

| Disease category | Sub-item |
| --- | --- |
| *I. Cardiovascular disorders* |  |
| Hypertension |  |
| Heart disease | Myocardial infarction |
|  | Recurrent chest pain on exertion |
|  | Angina pectoris |
|  | History of coronary bypass graft |
|  | History of coronary ballooning |
|  | History of pacemaker implantation |
|  | History of other cardiac surgery |
|  | Arrhythmia |
|  | Atrial fibrillation |
|  | Rheumatic heart disease |
|  | Valvular heart disease |
|  | Claudication |
|  | Other heart diseases |
| Thrombosis | Deep vein thrombosis |
|  | Pulmonary embolism |
|  | Other thrombosis |
| Other cardiovascular disease |  |
| *II. Central nervous system (CNS) disorders* |  |
| Stroke | Cerebral infarction |
|  | Cerebral hemorrhage |
|  | Transient ischemic attack |
|  | Other stroke |
| Parkinson’s disease |  |
| Multiple sclerosis |  |
| Epilepsy |  |
| Head trauma |  |
| Other CNS disease |  |
| *III. Endocrinologic disorders* |  |
| Hypercholesterolemia |  |
| Thyroid disease | Hyperthyroidism |
|  | Hypothyroidism |
|  | Other thyroid disease |
| Diabetes mellitus |  |
| Other endocrinologic disease |  |
| *IV. Neoplastic disorders* |  |
| *(asked to specify the history)* |  |
| Current treatment status |  |
| *V. Respiratory disorders* |  |
| Asthma |  |
| Chronic bronchitis |  |
| Pneumonia |  |
| Emphysema |  |
| Pulmonary tuberculosis |  |
| Hay fever (allergic rhinitis) |  |
| Other respiratory disease |  |
| *VI. Gastrointestinal disorders* |  |
| Gastritis |  |
| Gastroesophageal reflux disease |  |
| Hemorrhoid |  |
| Liver cirrhosis |  |
| Gallstone or biliary stone |  |
| Hepatitis |  |
| Irritable bowel syndrome |  |
| Constipation |  |
| Other gastrointestinal disease |  |
| *VII. Urogenital disorders* |  |
| Renal insufficiency |  |
| Benign prostate hyperplasia |  |
| Urinary tract infection |  |
| Cystitis |  |
| Other urogenital disease |  |
| *VIII. Musculoskeletal disorders* |  |
| Arthritis |  |
| Osteoporosis |  |
| Fracture |  |
| Other musculoskeletal disease |  |
| *IX. Infectious disorders* |  |
| Syphilis |  |
| AIDS |  |
| Other infectious disease |  |
| *X. Eye/ear disorders* |  |
| Cataract |  |
| Glaucoma |  |
| Otitis media |  |
| Other eye/ear disease |  |
| *XI. Psychiatric disorders* |  |
| Depression |  |
| Other psychiatric disease |  |
| *XII. Other major disease, accident or surgery* |  |
| (asked to specify the history) |  |
| *XIII. Admission history within recent 10 years* |  |
| (asked to specify the history) |  |
