# Supplementary material for: Cough in the Elderly Population: Relationships with Multiple Comorbidity
Source: PLoS One. 2013 Oct 21;8(10):e78081. doi: 10.1371/journal.pone.0078081 (PMC3804463; doi:10.1371/journal.pone.0078081)
Supplement: Table S2 — Logistic regression models for cough and comorbidity relationships among the subjects without abnormal chest radiographs and ACE inhibitor/codeine medication. (DOC) [file pone.0078081.s002.doc]

**Supporting information**

**Appendix Table**

Table S2. Logistic regression models for cough and comorbidity relationships among the subjects without abnormal chest radiographs and ACE inhibitor/codeine medication

|  | Frequent cough (n=65) vs. controls (n=544) |  | Chronic persistent cough (n=30) vs. controls (n=544) |  | Nocturnal cough (n=52) vs. controls (n=544) |  |
| --- | --- | --- | --- | --- | --- | --- |
|  | Adjusted OR (95% CI)* | *p* value | Adjusted OR (95% CI)* | *p* value | Adjusted OR (95% CI)* | *p* value |
| Age (years) | 0.96 (0.93–0.99) | 0.028 | 0.98 (0.93–1.03) | 0.347 | 0.92 (0.87–0.96) | 0.001 |
| BMI (kg/m^2^) | 0.95 (0.87–1.05) | 0.321 | 0.88 (0.77–1.01) | 0.060 | 1.05 (0.94–1.16) | 0.382 |
| Male gender | 0.41 (0.19–0.89) | 0.019 | 0.34 (0.12–1.00) | 0.050 | 0.45 (0.17–1.16) | 0.096 |
| Smoking status |  |  |  |  |  |  |
| Ex-smoker | 1.11 (0.42–2.93) | 0.837 | 1.01 (0.26–3.97) | 0.986 | 1.16 (0.38–3.56) | 0.800 |
| Current smoker | 3.92 (1.72–8.93) | 0.001 | 3.51 (1.20–10.3) | 0.022 | 1.49 (0.46–4.77) | 0.505 |
| Asthma | 3.98 (1.38–11.5) | 0.011 | 9.05 (2.95–27.8) | <0.001 | 7.24 (2.36–22.2) | 0.001 |
| Allergic rhinitis | 2.71 (1.01–7.29) | 0.048 | 5.87 (1.88–18.4) | 0.002 | 3.39 (1.24–9.28) | 0.018 |
| Diabetes mellitus | 2.65 (1.35–5.22) | 0.005 | 2.88 (1.10–7.55) | 0.031 | 1.40 (0.63–3.08) | 0.406 |
| HbA1C ≥ 8% | 7.98 (2.91–21.9) | < 0.001 | 8.23 (1.95–34.7) | 0.004 | 3.73 (1.16–12.0) | 0.028 |
| Constipation | 1.82 (0.85–3.91) | 0.124 | 4.93 (2.04–11.9) | < 0.001 | 2.81 (1.19–6.60) | 0.018 |
| Gastritis | 1.79 (0.84–3.83) | 0.132 | 2.65 (1.02–6.84) | 0.044 | 2.08 (0.94–4.63) | 0.072 |

Abbreviations: BMI, body mass index; OR, odds ratio; CI, confidence interval

For definitions, see Table 1 and 2.

*P* values were determined by multivariate logistic regression tests with adjustments for age, gender, BMI and smoking status
